# Genomic signatures and gene networking: challenges and promises

**DOI:** 10.1186/1471-2164-12-S5-I1

**Published:** 2011-12-23

**Authors:** Ke Zhang, Mehdi Pirooznia, Hamid R Arabnia, Jack Y Yang, Liangjiang Wang, Zuojie Luo, Youping Deng

**Affiliations:** 1 Department of Pathology, Bioinformatics Core, School of Medicine and Health Sciences, University of North Dakota, Grand Forks, ND 58201, USA; 2 School of Medicine, Johns Hopkins University, Baltimore, MD 21287, USA; 3 Department of Computer Science, University of Georgia, Athens, Georgia 30602-7404, USA; 4 Department of Radiation Oncology, Massachusetts General Hospital and Harvard Medical School, Boston, MA 02114, USA; 5 Department of Genetics and Biochemistry, Clemson University, Clemson, SC 29634, USA; 6 J.C. Self Research Institute of Human Genetics, Greenwood Genetic Center, Greenwood, SC 29646, USA; 7 Office of the President of The First Hospital, Guangxi Medical University, Nanning, Guangxi, China; 8 Department of Internal Medicine, Rush University Medical Center, Chicago, IL 60612, USA; 9 Rush University Cancer Center, Rush University Medical Center, Chicago, IL 60612, USA; 10 Department of Biochemistry, Rush University Medical Center, Chicago, IL 60612, USA

## Abstract

This is an editorial report of the supplement to BMC Genomics that includes 15 papers selected from the BIOCOMP'10 - The 2010 International Conference on Bioinformatics & Computational Biology as well as other sources with a focus on genomics studies.

BIOCOMP'10 was held on July 12-15 in Las Vegas, Nevada. The congress covered a large variety of research areas, and genomics was one of the major focuses because of the fast development in this field. We set out to launch a supplement to BMC Genomics with manuscripts selected from this congress and invited submissions. With a rigorous peer review process, we selected 15 manuscripts that showed work in cutting-edge genomics fields and proposed innovative methodology. We hope this supplement presents the current computational and statistical challenges faced in genomics studies, and shows the enormous promises and opportunities in the genomic future.

## 

Although the high throughput technology has made continuous progress during the last decade in terms of size, cost and signal quality, it remains challenging to deduce reliable predictive signatures from genomic data due to a small sample size and a large number of variables. Much effort of this supplement is devoted to developing predictive models for various types of genomic data, including mRNA, microRNA, and genome DNA. One of the important applications for RNA microarray data is to identify differentially expressed genes that can lead to gene signatures for predicting disease status and drug response. Mao *et al*. [1] investigated the differential gene expression between African Americans and Caucasians in white blood cells expression profiles for both type 2 diabetes patients and healthy people. The newly identified gene markers implicate the genetic basis for distinct risks of type 2 diabetes between these two populations. For microarray data, Due to the high cost of GeneChip, microarray experiments often have low sample sizes that present challenging for statistical analysis. In view of the difficulties for applying the common-used microarray analysis methods such as t-test, SAM, and FDR for small sample size experiment, Chen and his colleagues [2] proposed a model-based information sharing method (MBIS) that enhances the power of statistical test by utilizing information shared among genes. Next-generation sequencing technology enables the quantification of gene expression in the species whose gene chips are not available in market. Chen and his colleagues [3] used the 454 pyrosequencing technology to perform the transcriptome sequencing for an important herb medicine, the root of *Panax notoginseng*. This work discovered more than 20K unique transcripts and around 900 putative transcription factors.

When using gene signature for classification of disease phenotypes, it is critical to determine a subset of genes that is reliable across various studies and that provides high predictive power for the disease status. Liu *et al*. [4] have developed a gene selection algorithm, Recursive Feature Addition, that combines supervised learning method and statistical similarity measures. The gene signature was further optimized via a novel algorithm, Lagging Prediction Peephole Optimization. On the other hand, Shi and his colleagues [5] aimed to minimize the number of genes in a gene signature while maintaining its predictive power. They proposed a method called Minimize Feature's Size that makes use of similarity analyses between different endpoints and at multiple levels such as probe, gene, and GO. Both manuscripts validated their methods by comparing with various gene signature algorithms using benchmark microarray data.

The advocacy of personalized medicine in complicated diseases such as cancer and neural defects has made it increasingly important to identify genomic signatures that are associated with clinical outcomes. Several manuscripts of this supplement address questions in this aspect using various types of data. Zhao *et al*. [6] investigated a number of models for predicating cancer overall survival using gene expression profile from microarray data and found that the maximum predictive power of each model is limited by the correlation between endpoint and gene expression. Instead of looking at mRNA expression level, Zhang and his colleagues [7] focused on identifying DNA copy number variation that is correlated with cancer over survival. They developed a novel and efficient algorithm using a hidden Markov model to take into account the correlation between markers in SNP array. The algorithm classified glioma samples with distinct overall survival time. In the manuscript by Wang and his colleagues [8], they moved further to associate single nucleotide variations with single amino acid polymorphisms (SAPs) that can be used for predicting disease risk. They have validated their results using public datasets such as 1000 Genome Project and Genetic Analysis Workshop (GAW17). Protein-protein interaction (PPI) networks Protein functions were utilized by Huang and Chen [9] to predict drug cardiotoxicity. They proposed a systems biology framework to predict adverse drug reactions (ADR) using supervised learning methods such as support vector machine. This framework has a potential large impact to pharmaceutical industry for ADR is one of the major reasons for drug withdrawals in clinical trials.

Nowadays researchers are interested in not only what individual genes are activated, but also how genes interact with each other. Modelling gene networking presents a high challenge for bioinformatics because of incomplete information of gene functions and gene-gene interactions. About half of the manuscripts in this supplement are addressing questions regarding gene networking. Li *et al*. [10] developed a modified version of dynamic Bayesian Network for time-series microarray data, and showed that the proposed method provided an enhanced accuracy for predicting gene regulatory network structure. Wang and his colleagues [11] applied network analysis to protein-protein interaction data from the STRING database and identified a number of proteins that are associated with proteases Malaria parasite. These results illustrated the diverse functions of protease and implicated novel targets of drug design for Malaria.

DNA- or RNA-binding proteins play a critical role in gene regulatory networking. Liu and his colleagues [12] integrated the RNA sequence and secondary structures to to identify the consensus sequence of protein-RNA binding sites. This novel model-based approach, called RNAMotifModeler, demonstrated a number of statistical advantages when being applied to the RNA-binding protein SRSF1. The effect of epigenetic modification on gene regulation has been widely investigated. In this supplement, a manuscript by Zhao and his colleagues [13] looked into the combined regulation of epigenetic modification and miRNA in mediating gene networking. They conducted a genome-wide study and showed that DNA methylation and miRNA function are complementary to each other for gene regulation. This finding would advance our predictive models for gene regulatory networks by incorporating the epigenetic and miRNA factors.

Some of the authors devoted their efforts to traditional bioinformatics areas. Many alignment algorithm for DNA or protein sequences were proposed in 1980s, nonetheless, multiple sequence alignment is still a challenging question because of its computing intensity, which manifests with the advancement of the next-generation sequencing. Nguyen, Pan and Nong [14] provided a solution for multiple sequence alignment by combining the pair-wise dynamic programming algorithm with parallel computing approach using R-Mesh. This new method achieved computing time at *O(m)*, where *m *is the number of sequences. Bio-imaging analysis is another active field of bioinformatics. Tang and his colleagues [15] proposed a robust method to reduce the specles that present obstacles for ultrasound image post-processing. They used a detail preserving anisotropic diffusion filter and showed that their method prohibit over-diffusion and preserved the important structure information.

We are proud of the high quality of the manuscripts contained within this issue. We hope they would guide current genomics research and indicate the trend for future study.

### Acknowledgements

This supplement will not be possible without the support of the International Society of Intelligent Biological Medicine (ISIBM).
